# The Usefulness of Extended Inflammation Parameters and Systemic Inflammatory Response Markers in the Diagnostics of Autoimmune Hepatitis

**DOI:** 10.3390/cells11162554

**Published:** 2022-08-17

**Authors:** Weronika Domerecka, Anna Kowalska-Kępczyńska, Iwona Homa-Mlak, Agata Michalak, Radosław Mlak, Marcin Mazurek, Halina Cichoż-Lach, Teresa Małecka-Massalska

**Affiliations:** 1 Chair and Department of Human Physiology, Medical University of Lublin, 11 Radziwillowska Str., 20-080 Lublin, Poland; 2 Department of Biochemical Diagnostics, Chair of Laboratory Diagnostics, Medical University of Lublin, 20-081 Lublin, Poland; 3 Department of Gastroenterology with Endoscopy Unit, 8 Jaczewskiego Str., 20-090 Lublin, Poland

**Keywords:** autoimmune hepatitis, inflammation, extended inflammation parameters, systemic inflammatory response markers

## Abstract

(1) Introduction: Autoimmune hepatitis (AIH) is a chronic disease. A persistent autoimmune reaction in the liver is significantly related to the systemic inflammatory response. Extended Inflammation Parameters (EIP) can be used to assess the activation of immune cells such as activated neutrophils (NEUT-RI and NEUT-GI) and activated lymphocytes (RE-LYMP and AS-LYMP) in the phase of active inflammation. The role of the systemic inflammatory response markers should also be emphasised, especially: NLR, PLR, and RLR, which have recently been widely studied as markers in autoimmune skin diseases or liver diseases. (2) Materials and Methods: The study included 30 patients with AIH and 30 healthy volunteers. The parameters of the EIP group (RE-LYMP, AS-LYMP, NEUT-RI, NEUT-GI), calculated haematological indices Red Blood Cell Distribution Width-to-Platelet Ratio (RPR), Mean Platelet Volume-to-Platelet Ratio (MPR), Neutrophil-to-Lymphocyte Ratio (NLR), Platelet-to-Lymphocyte Ratio (PLR), Red Blood Cell Distribution Width-to-Lymphocyte Ratio (RLR), and selected blood morphological and biochemical indices were analysed. The aim of the study was to assess the usefulness of the EIP and systemic inflammatory response markers in the diagnostics of AIH. (3) Results: Compared to the controls, the patients with AIH showed significantly higher EIP values: NEUT-RI (48.05 vs. 43.30), NEUT-GI (152.65 vs. 147.40), RE-LYMP (0.07 vs. 0.03), and the inflammatory response markers: MPR (0.05 vs. 0.04), RPR (0.07 vs. 0.05), and NLR (2.81 vs. 1.42. Among the examined markers, EIP has significant diagnostic potential: NEUT-RI (AUC = 0.86), NEUT-GI (AUC = 0.80), and RE-LYMP (AUC = 0.78), and so do calculated haematological indices, i.e., MPR (AUC = 0.75), PLR (AUC = 1.00), and RLR (AUC = 1.00) Moreover, the importance of NEUT-GI (AUC = 0.89), MPR (AUC = 0.93), PLR (AUC = 0.86), RPR (AUC = 0.91), and FIB-4 (AUC = 0.83) in the detection of liver fibrosis in the course of AIH has also been proven. (4) Conclusions: EIP and systemic inflammatory response markers may turn out to be useful in detecting AIH and in looking for features of already developed liver cirrhosis in its course.

## 1. Introduction

Autoimmune hepatitis (AIH) is a chronic liver disease with an incidence of 11–25 people per 100,000 people in Europe [1]. A persistent autoimmune reaction in the liver is significantly related to the inflammatory response. Most often, the clinical picture is characterised by the slow onset and progression of the disease with nonspecific symptoms such as fatigue and malaise. Acute onset of AIH occurs in approximately one-third of patients. It can cause chronic hepatitis leading to cirrhosis (LC) [2]. AIH is more common in women than in men, as well as in children and the elderly (about 30% of all cases occur after the age of 60) [3]. The exact cause of AIH is unknown. It is believed that genetic, environmental (e.g., infections, drugs) and immunological (T-lymphocyte dysfunction) factors play an important role in the development of the disease [4]. AIH is diagnosed in patients with specific antibodies [antinuclear antibodies (ANA), liver/kidney microsome type 1 antibodies (anti-LKM1), anti-smooth muscle antibodies (SMA), as well as anti-soluble liver antigen/liver pancreas (anti-SLA)] and in those displaying elevated levels of immunoglobulin G (IgG) in their serum. As regards the antibody profile, AIH can be divided into several subtypes, namely type 1, type 2, type 3 AIH, and cryptogenic hepatitis. The diagnostic criteria of AIH are also based on the typical histologic demonstration (portal inflammation, interface hepatitis, and lobular hepatitis with varying severity). While treatment is most commonly based on corticosteroids and immunosuppression, biological drugs and cellular therapies can be also considered. For patients with features of severe liver failure (acute AIH) and for those with already developed LC and hepatocellular carcinoma, hepatic transplantation is recommended [5]. The total number of chronic liver disease (CLD) cases (with varying severity) equals 1.5 billion people across the world. Irrespective of the potential background of CLD (i.e., hepatitis B or C, non-alcoholic fatty liver disease, autoimmune diseases, alcohol-related disease, cholestatic disorders, and iron or copper overload), LC is known to be its final stage, constituting a significant cause of mortality and morbidity among patients. Of note is the fact that LC was found to constitute the eleventh leading cause of death, and even the fifteenth leading cause of morbidity, in the world. LC develops after a prolonged period of inflammation, finally transforming into a reversible replacement of the healthy hepatocytes with fibrotic tissue and regenerative nodules, which leads to the development of portal hypertension. The manifestation of LC concerns both its asymptomatic (compensated cirrhosis) and symptomatic stages (decompensated cirrhosis). Progressive portal hypertension, systemic inflammation, and liver failure add to a complex pathological profile of the disease. The management of LC focuses on disease causes and complications which, in some cases, may trigger the need to perform liver transplantation [6].

Recently, it has been possible to employ non-routinely used haematological parameters to find out about the activation status of the cells of the immune system in the laboratory diagnostics of inflammation. These parameters are available in the Sysmex Diagnostic haematology analysers as Extended Inflammation Parameters (EIP), which include such descriptors as: RE-LYMP (all activated lymphocytes), AS-LYMP (activated lymphocytes producing antibodies), and NEUT-RI and NEUT- GI (activated neutrophils). The RE-LYMP and AS-LYMP parameters provide information on the amounts of all reactive lymphocytes and antibody-secreting reactive lymphocytes in the peripheral blood. Lymphocyte populations are distinguished on the basis of their functionality and the resulting differences in the internal structure, the granularity present, and the size of the analysed cells. The parameters related to neutrophils, i.e., NEUT-RI and NEUT-GI, are indicative of the activation stage of the neutrophilic granulocytes. The measurement takes into account the metabolic activity of neutrophils, the internal structure and the size of the cell [7]. It was revealed that these parameters can be used in diseases of various aetiology, such as psoriasis [8], pemphigus [9], type II diabetes, and gynaecological diseases (endometriosis, uterine fibroids) [10].

In recent years, the role of systemic inflammatory response markers has also begun to be emphasised, in particular: the Neutrophil-to-Lymphocyte Ratio (NEU/LYM, NLR), the Platelet-to-Lymphocyte Ratio (PLT/LYM, PLR), the Red Blood Cell Distribution Width-to-Platelet Ratio (RDW/PLT, RPR), the Mean Platelet Volume-to-Platelet Ratio (MPV/PLT, MPR), and the Red Blood Cell Distribution Width-to-Lymphocyte Ratio (RDW/LYM, RLR) as potential diagnostic markers in various pathologies. It turned out that some of these can be used as markers in autoimmune skin diseases [11,12,13], in the course of neoplastic diseases [14,15], inflammatory bowel disease [16] or cardiovascular diseases [17]. Some reports have also shown their importance in the diagnosis of liver diseases [18,19,20]. These calculated haematological markers in patients with liver disease were usually viewed as potential markers of liver cirrhosis. The vast majority of studies investigate the roles of NLR and PLR in the decompensation of liver fibrosis or the development of hepatocellular carcinoma (HCC) due to the close relationship between liver pathologies and inflammation. NLR reflects the systemic inflammatory response, and the increase in its value corresponds to the increase in mortality in patients with LC [21]. This indicator is often cited as a prognostic factor, among others. In the course of acute coronary syndromes [17] or neoplasms [14,15], it has been suggested that an increased level of NLR is due to the release of pro-inflammatory cytokines. In the cellular reaction to an emerging damaging stimulus (e.g., a bacterial infection, trauma, chemical agents, or stress), an unspecific immune response is activated, along with the release of pro-inflammatory factors and an intensification of the inflammatory reaction. If this process is not inhibited after the primary damaging stimulus is removed, and the cells of the immune system are still highly overactive, it may result in the development of an autoimmune disease. As a result of the influence of pro-inflammatory factors, the activation of neutrophils, which have, for example, phagocytic abilities, is most likely to occur. At the same time, activated cytokine-releasing neutrophils and reactive oxidative species (ROS) can suppress the immune response of lymphocytes. It is believed that the activation of neutrophils, increased levels of IL-8, and other pro-inflammatory cytokines lead to more active NET formation. Prolonged exposure of the immune system to these processes increases the risk of developing autoimmune processes [22,23]. The number of neutrophils is, therefore, associated with the first non-specific inflammatory response of the body to the primary damaging stimulus or a prolonged abnormal response of the immune system to the stimulus already removed. In turn, the number of lymphocytes is related to the regulatory pathway of the immune system. A pathological appearance of LC is determined by the enhanced inflammatory response and mobilisation of immune cells. It is mostly connected with the increased synthesis of IL-6 and TNF-α. The underlying translocation of bacterial toxins from the gastrointestinal tract to the systemic circulation, because of portal hypertension, is directly followed by increased levels of neutrophils. Simultaneously, activated neutrophils may exert an inhibiting influence on the immune response generated by lymphocytes due to the production of arginase, nitric oxide and ROS. Additionally, lymphopenia might be the result of malnutrition among cirrhotic patients. The above-mentioned factors are inseparably involved in the higher results of NLR observed in the course of liver pathologies. Existing inflammation and the further development of portal hypertension in the natural history of AIH should result in elevated levels of NLR as well. However, trustworthy data devoted to this issue are still missing [24,25,26]. PLR was mostly explored among chronic HBV/HCV patients. Lower values of this parameter accompanied more advanced liver fibrosis, but the number of existing surveys is definitely small. However, high levels of PLR (together with NLR) were noted in patients with more advanced HCC and a greater recurrence risk [27]. So far, no significant differences in this parameter have been demonstrated between the AIH and control groups. The data concerning the role of PLR in the course of AIH are generally scant [26]. RLR appears to be explored so far among liver pathologies, only in primary biliary cholangitis (PBC) patients and people affected with acute hepatitis E virus (HEV) infection. RLR was higher in patients with HEV developing liver failure, compared with HEV-non-LF patients. The AUC value for RLR among the studied patients was 0.744. It was a marker of liver fibrosis with higher diagnostic accuracy compared to APRI, FIB-4, and RPR in the course of PBC and a marker of HEV infection in symptomatic patients [19]. However, the data on AIH and AIH-followed cirrhosis regarding the above-mentioned haematological indices are very limited. Of note, RPR was found to correlate with the severity of fibrosis in the course of AIH [28,29].

Nevertheless, these indicators have so far only been the subject of scientific research and not an element of everyday clinical practice. Considering the above examples, the above-mentioned markers seem to be potentially and particularly useful in the diagnosis of sudden liver decompensation in the course of chronic liver failure.

In the laboratory diagnostics of AIH, the determination of biochemical markers of inflammation is used, e.g., C-reactive protein (CRP) concentration. CRP is an acute-phase protein that is mainly produced by hepatocytes in response to inflammation. It serves as a marker of systemic inflammation, where it is involved in the opsonisation and activation of the complement system in response to the secretion of pro-inflammatory cytokines. High serum CRP levels are associated with colorectal cancer and other cancer types. Among cancer patients, CRP also plays a role as a prognostic marker in ovarian, oesophageal, and gastric cancer. Recent data suggest that CRP may be a prognostic factor for liver cancer and cirrhosis [30].

To the best of our knowledge, there are no studies on the role of EIP markers and the systemic inflammatory response markers in AIH. Few studies discuss the role of non-invasive enumerated inflammatory markers in predicting the degree of LC in patients with AIH. Taking into account the systemic nature of AIH, it seems important to search for diagnostic indicators which will be useful in the detection of an increased systemic inflammatory response, which is inextricably linked to the course of this disease. Due to the presence of inflammation as a key element of AIH, we decided to verify the role of EIP and the systemic inflammatory response markers in the diagnostics of this disease.

## 2. Material and Methods

### 2.1. Characteristics of Patients

In total, 60 participants were enrolled in the study: 30 patients with AIH representing the study group (27 women and 3 men over the age of 18) and 30 healthy persons in the control group (25 women and 5 men) (*p* = 0.4518). The median age in the study group was 56 years and in the control group it was 43 years (*p* = 0.1313). The study and control groups were also well balanced in terms of BMI, smoking status, and excessive alcohol consumption (*p* = 0.1391, *p* = 0.2424, and *p* = 0.2857, respectively). Commonly known guidelines (the presence of typical antibodies, an increased level of immunoglobulin G, and a typical histologic demonstration in liver histology) were used to establish the diagnosis of AIH [2]. Other chronic or acute liver pathologies were excluded. No viral, cholestatic liver disorders or clinically significant inflammatory processes were observed in any of the survey participants. In 10 AIH patients, LC was already diagnosed based on common criteria [5]. The Doppler-mode abdominal ultrasound examination was used to visualise the presence of portal hypertension (the diameter of portal vein ≥ 13 mm), with additional potential factors responsible for the development of existing portal hypertension being excluded. The local Ethics Committee of the Medical University of Lublin approved the study (No. KE-0254/21/2016), and written informed consent forms were signed by all patients, in accordance with the Helsinki Declaration, for the procedures they underwent.

The clinical and demographic characteristics of the study participants are presented in Table 1.

### 2.2. Apparatus and Methodology

The haematological determinations were performed using the Sysmex XN 1500 apparatus (Sysmex Europe SE, Warsaw, Poland), and the biochemical determinations were performed using the Cobas 6000 apparatus (Roche Diagnostics Polska, Warsaw, Poland).

The research material was blood obtained from a vein in the arm. Blood sampling was performed in the morning in fasting patients. First, blood samples were drawn to clot vacuum tubes sized 8–16 × 50–100 mm, in the amount of approx. 7.6 mL, in order to determine biochemical parameters. Then, additional blood samples were taken to 2.7 mL vacuum tubes containing the anticoagulant K3EDTA (tripotassium ethylenediaminetetraacetic acid, Sarstedt) in order to be able to determine the concentrations of the selected haematological parameters. The blood in the clot tubes was allowed to clot for about 20–30 min, and then it was centrifuged at a speed of 2500 rpm for 10 min. The whole blood for haematology tests, collected on K3EDTA, was analysed up to 1 h after receiving the material.

The following parameters were analyzed: Reactive Lymphocytes (RE-LYMP, expressed as an absolute number in [103/µL]), Antibody-Secreting Reactive Lymphocytes (AS-LYMP, expressed as an absolute number in [103/µL]), Neutrophil Reactive Intensity (NEUT-RI, expressed in [FI] units, describing the fluorescence intensity), Neutrophil Granularity Intensity (NEUT-GI, expressed in [SI] units, describing the light intensity of the scattered laser beam), White Blood Cells (WBC, [K/µL]), Red Blood Count (RBC, [M/µL]), Platelets (PLT, [K/µL]), Neutrophils (NEUT, [K/µL]), Lymphocytes (LYMP [K/µL]), Monocytes (MONO, [K/µL]), Immature Granulocytes (IG, K/µL], Red Blood Cell Distribution Width Standard Deviation (RDW-SD, [fl]), Mean Platelet Volume (MPV, [fl]), and Mean Cell Volume (MCV, [fl]). The tested haematological indexes, based on the basic blood count parameters, were: the Neutrophil-to-Lymphocyte Ratio (NEU/LYM, NLR), the Platelet-to-Lymphocyte Ratio (PLT/LYM, PLR), the Red Blood Cell Distribution Width-to-Platelet Ratio (RDW/PLT, RPR), the Mean Platelet Volume-to-Platelet Ratio (MPV/PLT, MPR), and the Red Blood Cell Distribution Width-to-Lymphocyte Ratio (RDW/LYM, RLR), while the assessed biochemical parameters were the levels of C-reactive Protein (CRP [mg/L]), Alanine Aminotransferase (ALT, [IU/L]), Aspartate Aminotransferase (AST, [IU/L]), Alkaline Phosphatase (ALP, [IU/L], Gamma-Glutamyl Transpeptidase (GGTP, [IU/L]), Bilirubin [mg/dL], the Aspartate Aminotransferase-to-Alanine Aminotransferase Ratio (AST/ALT, AAR), the Aspartate Aminotransferase-to-Platelet Ratio Index (AST/PLT, APRI), Fibrosis-4 (age * AST/PLT * ALT ½, FIB-4), and the Gamma-Glutamyl-Transpeptidase-to-Platelet Ratio (GGTP/PLT, GPR).

### 2.3. Statistical Methods

The collected data were analysed using Statistica v. 13 PL (Cracow, Poland) and MedCalc v 15.8 PL software (Ostend, Belgium). The distribution of the categorised data is presented as percentages. The normality of the distribution of continuous data was assessed using the D’Agostino–Pearson test. For non-normal distributions, non-parametric tests and medians, as well as interquartile ranges, were used as measures of data clustering and dispersion, respectively. In order to assess the differences between the continuous variables, the Mann–Whitney U test was used. The correlation between the variables was assessed using the Spearman’s rank correlation test. In the assessment of the diagnostic usefulness of the selected variables (for which statistically significant results were obtained in the Mann–Whitney U test, or if these were parameters not assessed routinely), ROC curve analysis was used to differentiate distinct clinical conditions. Two-tailed tests were used in all analyses, and the results of *p* < 0.05 were considered statistically significant.

## 3. Results

### 3.1. Extended Inflammation Parameters and Systemic Inflammatory Response Markers and Serous Indirect Markers of Liver Fibrosis in Diagnostics of Autoimmune Hepatitis

**Figure 1:**
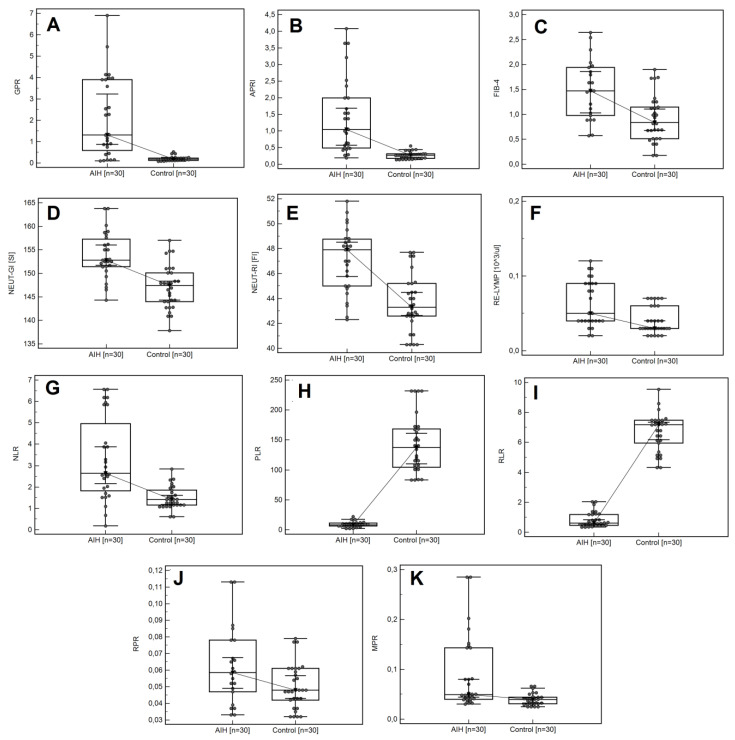
Box-whisker plot comparing the values of: GPR (**A**), APRI (**B**), FIB-4 (**C**), NEUT-GI (**D**), NEUT-RI (**E**), Re-LYPMH (**F**), NLR (**G**), PLR (**H**), RLR (**I**), RPR (**J**), and MPR (**K**) in the AIH group and the control group.

Compared to people in the control group, the patients with AIH showed higher median values of standard haematological parameters: MCV (92.55 vs. 87.2 [fl]; *p* < 0.0001), RDW-SD (46.4 vs. 41.27 [fl]; *p* = 0.0013), MPV (11.3 vs. 10.1; *p* = 0.0001), WBC (6.47 vs. 5.58 [10^3^/µL]; *p* = 0.0147), NEUT (4.53 vs. 2.74 [10^3^/µL]; *p* = 0.0002), IG (0.03 vs. 0.01 [10^3^/µL]; *p* = 0.0001), parameters from the EIP group: NEUT-RI (48.05 vs. 43.3 [FI]; *p <* 0.0001), NEUT-GI (152.65 vs. 147.4 [SI]; *p =* 0.0001), and RE–LYMP (0.07 vs. 0.03 [10^3^/µL]; *p =* 0.0001). Significantly higher median systemic inflammatory response markers were found in the patients with AIH compared to the controls: NLR (2.81 vs. 1.42; *p <* 0.0001), RPR (0.07 vs. 0.05; *p =* 0.0007), and MPR (0.05 vs. 0.04; *p =* 0.0004). In the patients with AIH compared to the control group, statistically significantly higher medians for standard biochemical parameters were also noted: CRP (3 vs. 1.5 [mg/l]; *p* = 0.0043), AST (54 vs. 21.5 [IU/L]; *p* < 0.001), ALT (62.5 vs. 18. [IU/L]; *p* < 0.0001), Bilirubin (1.5 vs. 0.6 [mg/dl]; *p* = 0.0002), GGTP (100.50 vs. 18.00 [IU/L]; *p* < 0.0001), and ALP (128 vs. 68 [IU/L]; *p* = 0.002). Similar differences were noted for serous indirect markers of liver fibrosis: GPR (1.78 vs. 0.18; *p* < 0.0001), APRI (1.37 vs. 0.27; *p* < 0.0001), and FIB-4 (1.82 vs. 0.83; *p* < 0.0001). Detailed data, including a comparison of the study and control groups with regard to selected laboratory parameters, are presented in Supplementary Table S1, Figure 1A–K.

**Figure 2:**
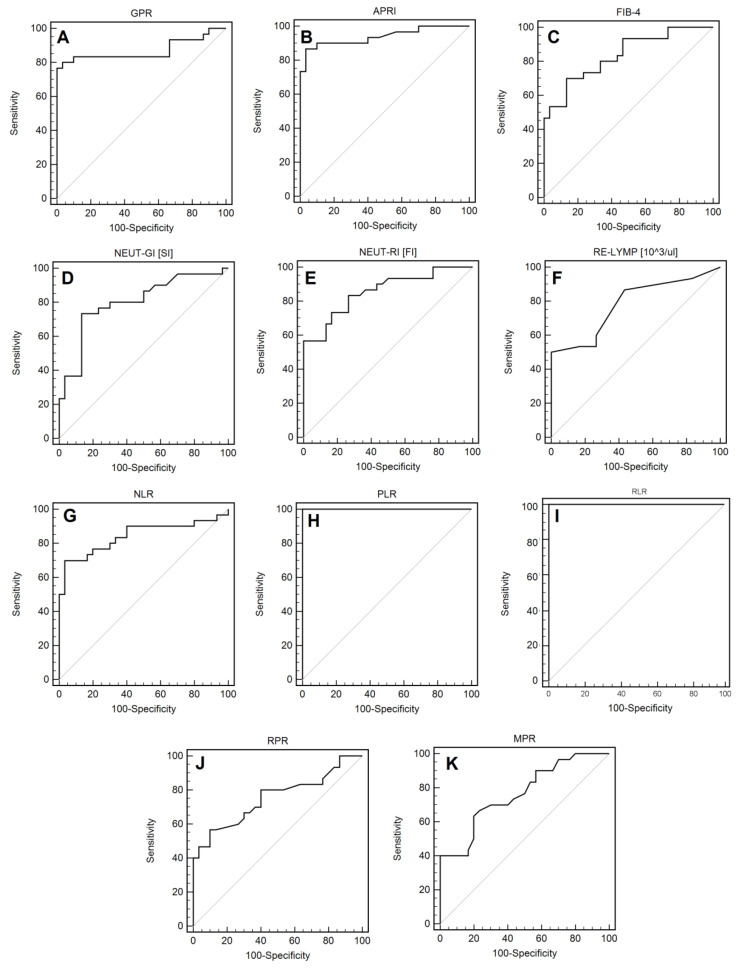
ROC curves representing the assessment of the diagnostic usefulness of the values of GPR (**A**), APRI (**B**), FIB-4 (**C**), NEUT-GI (**D**) NEUT-RI (**E**), RE-LYMP (**F**), NLR (**G**), PLR (**H**), RLR (**I**), RPR (**J**), and MPR (**K**) in the detection of AIH.

Among the standard haematological parameters, the following parameters were of significant and high diagnostic usefulness in detecting AIH: MCV [fl] (sensitivity 76.67%, specificity 85% (AUC = 0.80; *p* < 0.0001), PLT [10^3^/µL] (sensitivity 40%, specificity 100%; AUC = 0.80; *p* < 0.0001), RDW-SD [fl] (sensitivity 60%, specificity 100%; AUC = 0.84; *p* < 0.0001), MPV [fl] (sensitivity 86.67%, specificity 60%; AUC = 0.79; *p* < 0.0001), NEUT [10^3^/µL] (sensitivity 80%, specificity 76.67%; AUC = 0.78; *p* < 0.0001), LYMPH [10^3^/µL] (sensitivity 63.33%, specificity 96.67%; AUC = 0.78; *p* < 0.0001), NEUT-RI [FI] (sensitivity 83.33%, specificity 73.33%; AUC = 0.86; *p* < 0.0001), NEUT-GI [SI] (sensitivity 73.33%, specificity 86.67%; AUC = 0.80; *p* < 0.0001), and RE-LYMP [10^3^/µL] (sensitivity 52%, specificity 100%; AUC = 0.78; *p* < 0.0001). Among the calculated systemic inflammatory response markers, the PLR and RLR parameters were characterised by the highest, as much as 100%, sensitivity and specificity (AUC = 1.00; *p* < 0.0001) in detecting AIH. The following indicators were characterised by lower sensitivity, but high specificity: RPR (sensitivity 56.67%, specificity 90%; AUC = 0.75; *p =* 0.0001), NLR (sensitivity 70%, specificity 96.67%; AUC = 0.84; *p* < 0.0001), and MPR (sensitivity 66.67%, specificity 76.67%; AUC = 0.77; *p* < 0.0001). Among the serous indirect markers of liver fibrosis, the most useful were GPR (sensitivity 80%, specificity 96.67%; AUC = 0.87; *p <* 0.0001), APRI (sensitivity 86.67%, specificity 96.67%; AUC = 0.94; *p <* 0.0001), and FIB-4 (sensitivity 70%, specificity 86.67%; AUC = 0.84; *p <* 0.0001). The detailed data are included in Table 2 and Figure 2A–K.

### 3.2. The Correlation between EIP and Systemic Inflammatory Response Markers and Serous Indirect Markers of Liver Fibrosis in Study Group (AIH)

Statistically significant negative correlations were observed between: NEUT-GI and MPR (rho = −0.493; *p* = 0.0056), and RPR (rho = −0.477; *p* = 0.0077) and FIB-4 (rho = −0.659; *p* = 0.0001). The detailed data on the dependences between the EIP and systemic inflammatory response markers together with the serological markers of liver fibrosis in the study group (AIH) are presented in Supplementary Table S2.

### 3.3. The Correlation between EIP and Systemic Inflammatory Response Markers and Serous Indirect Markers of Liver Fibrosis in Both the AIH-Non-LC Group and AIH-LC Group

In the AIH subgroup without features of cirrhosis, RE-LYMP correlated positively with PLR (rho = 0.641; *p* = 0.0013). A statistically significant, negative correlation was observed between the NEUT-GI and FIB-4 markers (rho = −0.519; *p* = 0.0133). The detailed data on the evaluated dependences between the EIP and systemic inflammatory response markers together with serological parameters of liver fibrosis in the AIH-non-LC group are presented in Supplementary Table S3.

No statistically significant correlations in the AIH-LC group were noted. The detailed data on the evaluated dependences between the EIP and systemic inflammatory response markers, together with serological parameters of liver fibrosis in the study group (AIH), are presented in Supplementary Table S4.

### 3.4. Assessment of the Diagnostic Usefulness of Selected Laboratory Parameters in the Differentiation of LC (Liver Cirrhosis) and Non-LC (Non-Liver Cirrhosis) in AIH

**Figure 3:**
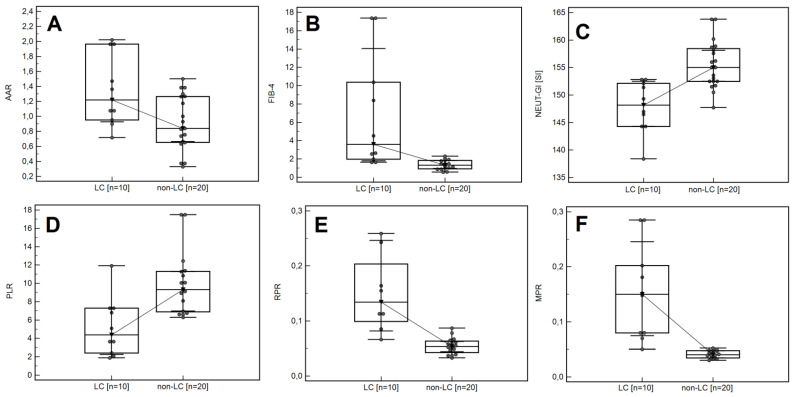
Box-whisker plot comparing the values of AAR (**A**), FIB-4 (**B**), NEUT-GI (**C**), PLR (**D**), RPR (**E**), and MPR (**F**) in the LC group and the non-LC group.

Significantly higher median values of standard haematological parameters were noted in patients with no cirrhosis compared to the group of patients with cirrhosis in AIH: PLT (241.5 vs. 85 [10^3^/µL]; *p =* 0.0001),WBC (7.75 vs. 4.94 [10^3^/µL]; *p =* 0.0002), NEUT (4.91 vs. 3.52 [10^3^/µL]; *p =* 0.0114), LYMPH (1.54 vs. 0.86 [10^3^/µL]; *p =* 0.0005), MONO (0.66 vs. 0.43 [10^3^/µL]; *p =* 0.0114), IG (0.04 vs. 0.02 [10^3^/µL]; *p =* 0.0197), and NEUT-GI (155 vs. 148.15 [SI]; *p =* 0.0006). There was a significantly higher median of systemic inflammatory response markers in patients without cirrhosis compared to those with cirrhosis: PLR (10.06 vs. 4.37; *p =* 0.0017). Compared to patients with LC, the non-LC group recorded significantly lower medians of the selected serous indirect markers of liver fibrosis: AAR (0.88 vs. 1.22; *p* = 0.0408) and FIB-4 (1.46 vs. 3.59; *p* = 0.0034), and the systemic inflammatory response markers: MPR (0.04 vs. 0.15; *p* = 0.0002) and RPR (0.06 vs. 0.16; *p* = 0.0003). The detailed data, including a comparison of selected morphological and biochemical parameters depending on the presence of LC and non-LC, are included in Supplementary Table S5, Figure 3A–F.

**Figure 4:**
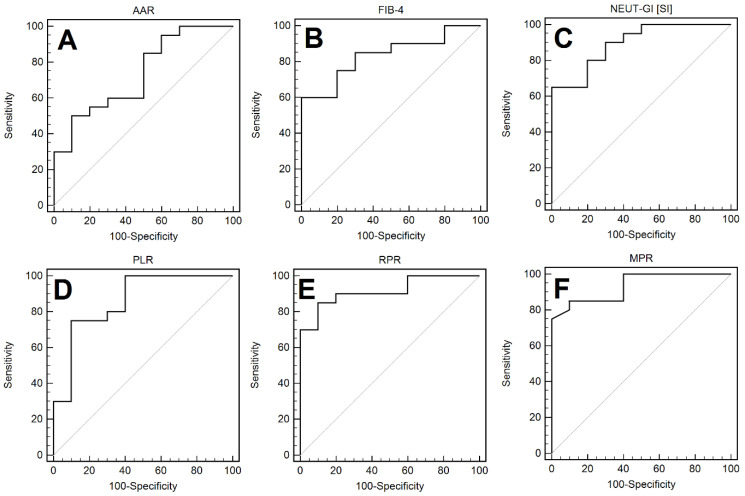
ROC curves representing the assessment of the diagnostic usefulness of the AAR (**A**), FIB-4 (**B**), NEUT-GI (**C**), PLR (**D**), RPR (**E**), and MPR (**F**) in the detection of LC in the course of AIH.

The AUC values, along with the determined cut-off points and the evaluation of the sensitivity and specificity of selected laboratory parameters in the detection of LC in the course of AIH, are presented in Table 3 and Figure 4A–F. The analysis of the results of the group of patients with AIH showed that the most useful in differentiating LC and non-LC, among the standard haematological parameters, were PLT (sensitivity 80%, specificity 100%; AUC = 0.94; *p* < 0.0001), WBC (sensitivity 80%, specificity 100%; AUC = 0.99; *p* < 0.0001), NEUT (sensitivity 50%, specificity 100%; AUC = 0.78; *p* = 0.0069), LYMPH (sensitivity 100%, specificity 80%; AUC = 0.90; *p* < 0.0001), MONO (sensitivity 55%, specificity 100%; AUC = 0.79; *p* = 0.0006), and NEUT-GI (sensitivity 65%, specificity 100%; AUC = 0.89; *p* < 0.0001). Among the systemic inflammatory response markers, MPR (sensitivity 75%, specificity 100% (AUC = 0.93; *p <* 0.0001) PLR (sensitivity 75%, specificity 90%; AUC = 0.86; *p <* 0.0001) and RPR (sensitivity 85%, specificity 90%; AUC = 0.91; *p =* 0.0001) were characterised by high diagnostic usefulness in differentiating LC and non-LC patients. Among serous indirect markers of liver fibrosis, this was FIB-4 (sensitivity 60%, specificity 100%; AUC = 0.83; *p <* 0.0001).

### 3.5. Comparison of Selected Laboratory Variables, including Extended Inflammation Parameters as well as Calculated Indicators—Systemic Inflammatory Response Markers and Serous Indirect Markers of Liver Fibrosis Depending on Applied Treatment

In the patients with AIH treated with steroids alone [B], a combination of steroids and immunosuppressive agents [C], or subjected to any of the above treatment schemes [D] compared to those in whom no drug was used [A], we noted significantly lower medians of NEUT-RI (EIP parameter) (47.90 or 47.45 or 47.00 vs. 52.95 [Fl]; *p* = 0.0240 or *p* = 0.0062 or *p* = 0.0088). Similarly, in the patients with AIH treated with a combination of steroids and immunosuppressive agents [C] or subjected to any of the above treatment schemes [D] compared to those in whom no drug was used [A], we noted significantly lower medians of AAR (an indirect marker of liver fibrosis) (0.96 or 0.91 vs. 1.41; *p* = 0.0196 or *p* = 0.0262). The detailed data on the comparison of selected laboratory variables, including Extended Inflammation Parameters as well as calculated indicators—systemic inflammatory response markers and serous indirect markers of liver fibrosis depending on applied treatment are presented in Supplementary Table S6.

## 4. Discussion

In recent years, the potential role of EIP and the systemic inflammatory response markers in the context of the diagnosis of liver and other organs or tissue pathology, in particular inflammatory diseases, has significantly grown in importance. However, most of them concern viral liver damage [31], alcoholic hepatitis [32], or autoimmune skin diseases [11,12,13].

The aim of this study was to assess the diagnostic usefulness of selected EIP and systemic inflammatory response markers in the diagnosis of AIH. The results of the conducted research lead to the conclusion that, in detecting AIH, apart from the basic, routinely used parameters, selected EIP (NEUT-RI, NEUT-GI, and RE-LYMP) or systemic inflammatory response markers (MPR, PLR, RPR, RLR, and NLR) were found to be useful. It turned out that, in this respect, the highest diagnostic usefulness (AUC > 0.90) in detecting AIH, in addition to the standard AST and ALT parameters, was exhibited by the following parameters: MPR, PLR, and RLR. High diagnostic usefulness (AUC > 0.80), apart from the standard MCV, RDW-SD, ALP, and GGTP, was also displayed by NEUT-RI, NEUT-GI, and NLR, while good diagnostic usefulness (AUC > 70) was characterised, apart from the typically assessed PLT, MPV, IG, and CRP, with the following parameters: RE-LYMP, MPR, and RPR. We also assessed the diagnostic usefulness of EIP and systemic inflammatory response markers in differentiating LC and non-LC patients with AIH. It turned out that, in this respect, the highest diagnostic usefulness (AUC > 0.90) was noted, apart from the typically assessed WBC, PLT, and LYMPH, for the following parameters: NEUT-GI, MPR, and RPR, and high diagnostic usefulness (AUC > 0.80), apart from the routinely assessed MONO also for PLR, while in the study group, there was only one standard parameter displaying good diagnostic usefulness (AUC > 0.70), i.e., the standard NEUT parameter.

Many studies have been carried out on the potential role of the EIP and systemic inflammatory response markers (e.g., PLR, RPR, and NLR) in a number of chronic diseases where the inflammatory component plays an important role. It turned out that some of them (e.g., NLR, PLR, and RPR) can be used as markers useful in the diagnosis of neoplastic diseases [14,15], inflammatory bowel diseases [16] or cardiovascular diseases [17]. Some reports have also shown their usefulness in the diagnosis of liver diseases (e.g., NLR, PLR, RLR, and RPR). These calculated haematological parameters in patients with liver disease were usually viewed as potential markers of cirrhosis [25,33]. Nevertheless, to the best of our knowledge, they have not yet been studied as useful markers in the diagnosis of AIH.

The increased activation of immune system cells in the course of AIH, as was found in our study, in the form of a higher median value of the NEUT-RI, NEUT-GI, and RE-LYMP parameters in people with AIH, compared to the control group, seems to be a consequence of an inflammatory process in the liver, which is characterised by the infiltration of inflammatory cells within hepatocytes [34]. These parameters, together with clinical data, may constitute a useful auxiliary tool in the diagnosis of the development of an inflammatory reaction in the course of AIH. The available knowledge and literature data show that changes in these parameters are also observed in the disease entities of a different aetiology. These changes, consisting of an increase in the levels of EIP parameters, were observed, for example, in the course of bacterial and viral infections. Henriot et al. [35] examined whole blood from three groups of patients for changes in EIP: (1) a group of healthy people, (2) patients diagnosed with diseases of a bacterial aetiology (pyelonephritis or gastroenteritis), and (3) patients with viral diseases (upper respiratory tract infections, meningitis, EBV, CMV, and the chickenpox infection). The results of these studies, as well as our results, showed significant changes to the parameters belonging to EIP. In the course of bacterial infection, a significant increase in the NEUT-RI parameter was observed, while in the case of viral infections, the increase concerned mainly lymphocytic parameters (RE-LYMP and AS-LYMP). Henriot et al. emphasised the similarity of the power to distinguish bacterial from viral aetiology thanks to the use of EIP (AUC = 0.83) compared to procalcitonin (AUC = 0.82) [35]. Park et al. [36], who assessed the new haematological parameters in sepsis patients with confirmed Gram-positive, Gram-negative, or mixed bacteraemia, showed that neutrophilic parameters (NEUT-RI, NEUT-GI) displayed similar or higher values describing diagnostic usefulness (AUC = 0.909 and AUC = 0.905, respectively) compared to routinely performed markers of inflammation such as white blood cells, neutrophils, lymphocytes and haemoglobin levels. Similar parameters of neutrophilic parameters for the course of sepsis were obtained by carrying out haematological examinations in patients suffering from neoplastic disease (lung cancer, nasopharyngeal carcinoma, colorectal cancer, liver cancer, gastric carcinoma, head and neck neoplasm, oesophageal cancer, pancreatic cancer, prostate cancer, ovarian cancer, pelvic carcinoma, renal carcinoma, bladder cancer, cervical cancer, teratoma, and breast cancer) [37]. These studies have shown that the NEUT-RI and NEUT-GI parameters in this group of patients correlate positively (with a mean correlation strength) with the level of procalcitonin (r = 0.530, *p* = 0.002). In turn, the research conducted by our team has shown that the parameter describing the activation of lymphocytes (RE-LYMP) increases significantly with the severity of the autoimmune disease, such as psoriasis.

This parameter strongly and positively correlated with the severity of the disease expressed in the PASI (r = 0.769, *p* = 0.045) and BSA (r = 0.703, *p* = 0.045) scales [8]. The above-mentioned results of the work, as well as our research described in this publication, show the huge diagnostic potential of EIP. These parameters have recently been intensively studied because their changes may allow the determination of the severity of inflammation in various immune-mediated diseases such as AIH.

Following the conclusions described in the above-mentioned studies, we also analysed and compared the diagnostic values of haematological parameters and selected blood count indicators for the detection of liver fibrosis. It turned out that, in this respect, the determination of parameters such as WBC, PLT, NEUT, LYMPH, MONO, NEUT-GI, MPR, PLR, and RPR is of great importance. The PLR parameter is the subject of research in the field of hepatology. So far, scientific reports have shown that it may positively correlate with the probability of HCC recurrence after LT. In total, 899 patients were examined, 80% of whom were male. In all studies except one, a PLR cut-off of 150 was determined. In the meta-analysis, a high PLR value was associated with an over three-fold risk of HCC recurrence after LT (OR = 3.33; 95% CI: 1.78–6.25; *p* < 0.001). Similar observations were noted for the NLR index. Elevated pre-transplant NLR was closely related to the overall rate of survival (HR: 2.22; 95% CI: 1.34–3.68) and relapse-free survival (HR: 3.77; 95% CI: 2.01–7.06) in patients undergoing LT due to HCC [38]. It has been shown that a higher level of NLR and a higher concentration of CRP determine a much worse prognosis and are associated with higher mortality rates in patients with LC [25,33]. Thus, the phenomenon of an increased systemic inflammatory response is inseparable from the course of liver function decompensation. The number of neutrophils can be related to the inflammatory response of the body, and the number of lymphocytes can be related to the regulatory pathway of the immune system. It was also noticed that higher PLR and NLR values may play a role in neoplastic diseases and accompany pancreatic and bile duct cancers and be predictors of their worse prognosis. The increase in these parameters reflects the inflammation associated with neoplastic growth [39,40]. In their manuscript, Recio-Boiles et al. analysed data from 55 patients diagnosed with metastatic pancreatic ductal adenocarcinoma (PDAC). The NLR and PLR values were calculated on the day of the diagnosis and surgery. The aim of this study was to correlate NLR and PLR with the radiological clinical stage and the biochemical tumour marker (CA 19–9). They showed that NLR and PLR can predict the prognosis and extent of the tumour in these patients. Interestingly, they presented that preoperative NLR and PLR at diagnosis correlated inversely with resection margins (R) and the lymph nodes (LN) status. The NLR/PLR for R0, R1, and R2 were 6.7/241, 4.8/224, and 2.9/147, respectively (*p* = 0.01/0.002); consequently, the NLR/PLR for N0, N1, and N2 were 5.1/212 and 2.7/138.3, respectively (*p* = 0.03/0.009). The majority of the already performed studies proposed NLR and PLR as indicators of the progressing neo-plasmatic process. These discrepancies require further studies [39]. Kitano et al., while examining a group of 120 patients who underwent surgery for extrahepatic biliary carcinoma (ECC), assessed the rates of the systemic inflammatory response, i.e., NLR and PLR, before and after the surgery. Their results showed that high preoperative PLR was an independent, unfavourable prognostic factor in resected ECC patients [40]. Zeng et al. did not find a significant difference in the PLR values in AIH patients compared to the controls [26].

It has already been found that haematological indicators, e.g., RLR, RPR, and RDW, are potential markers of LC progression [18,19,20] and further HCC development [38]. RLR is a new potential predictor of the liver fibrosis process. Meng et al. examined a group of 94 patients (74 patients with PBC and early LC, 20 patients with PBC and advanced LC). Patients in the advanced stage of the disease showed significantly higher RPR (*p* = 0.003), RLR (*p* = 0.001), APRI (*p* = 0.001) and FIB-4 (*p* = 0.003) compared to early-stage patients. The authors also showed good diagnostic usefulness of RLR (sensitivity 65.00%, specificity 78.38%; AUC = 0.744), which was greater than that of APRI (sensitivity 75.00%, specificity 75.68%; AUC = 0.742), FIB-4 (sensitivity 80.00%, specificity 71.62%; AUC = 0.718), and RPR (sensitivity 55.00%, specificity 86.49%; AUC = 0.719), They thus proved that this marker can be useful in predicting LC advancement in the course of PBC [18]. Another work dedicated to this indicator concerned the population of people with HEV infection. The study included 93 controls, 152 patients with early diagnosis of HEV infection without hepatic insufficiency (HEV-non-LF) and 62 HEV patients who developed hepatic failure by (HEV-LF). The investigators demonstrated good diagnostic usefulness of the RLR in predicting HEV-related liver failure (sensitivity 74%, specificity 65%; AUC = 0.74) and it was higher than the NLR (sensitivity 66%, specificity 70%; AUC = 0.72) and RDW (sensitivity 58%, specificity 67%; AUC = 0.63) [19]. The data obtained in a study conducted by He et al., involving 84 controls, 52 patients with chronic HCV and 42 patients with HCV-associated cirrhosis, also support a significant role of RDW and RPR. They achieved statistically significantly higher values in the group of patients with developed LC compared to those with chronic C hepatitis (CHC) (RDW: 16.44 ± 3.01 vs. 14.12 ± 1.54; RPR: 0.28 ± 0.15 vs. 0.09 ± 0.04; *p* < 0.001). The increase in the value of the RDW index was also an independent predictor of LC development in this study, as was the case with autoimmune liver damage. These parameters were also very useful in the diagnosis of liver fibrosis: RDW (AUC = 0.791) and RPR (AUC = 0.960) [20].

However, these markers have not been implemented as routine parameters in hepatology. There is still a great need for new research in this field of medicine. The presented study tries to fill this gap. Following the data from the literature, it seems that the above-described relationships between the systemic inflammatory response (i.e., RPR, MPR, PLR, WBC, PLT, MPV, NEUT, LYMPH, and MONO) and EIP, and the development of AIH, including LC, have not been published so far and are the first reports in this area. The literature was searched for using the PubMed database, after entering such words as: EIP, RE-LYMP, NEUT-GI, NEUT-RI, AIH, LC, NLR, PLR, and MPR. However, there are still some limitations that should be highlighted. We examined a relatively small population of AIH patients and did not associate haematology with liver biopsy samples. Nevertheless, this was a pilot study and further research will not only look at blood test results but will also assess the potential relationship between new markers of inflammation in the blood and the severity of inflammation in the liver biopsies of patients with AIH.

The increased levels of NEUT-GI in AIH patients compared to the AIH-followed LC group suggest the significance of inflammation as a background for the observed differences in the values. LC is a final stage of various hepatic pathologies and is characterised by relatively low degrees of inflammation, being a kind of burned-out disorder. The remaining calculated indicators have not been tested in the context of AIH so far.

AIH patients investigated in the study were in the phase of clinical and laboratory remission. We did not perform liver biopsies on them. To verify the potential impact of steroids and immunosuppressant intake on the results of the assessed inflammatory markers, we compared the AIH subgroups with each other (steroids vs. immunosuppressants vs. steroids + immunosuppressants). Of note, we found significant differences according to NEUT-RI and AAR. The values of these parameters were significantly lower in AIH patients treated pharmacologically. It is quite an interesting observation because of the chronic and maintenance character of immunosuppressive therapies in patients included in the research group. These were long-lasting therapies existing through the years (with the median of the disease duration of 13 years), therefore it is hard to suspect with certainty the presence of a potential relationship between the treatment and the character of the results in the examined patients. Nevertheless, this issue requires further exploration, data obtained from liver biopsy, and the investigation of AIH patients with an active type of the disease, as well. According to the available literature, we seem to present such data for the first time. Probably, chronic immunosuppressive therapy was a factor which caused a decrease in hepatitis’ severity and it was reflected by lower values of NEUT-RI and AAR. Thus, it appears that NEUT-RI could be explored as a potential valuable marker differentiating AIH patients with and without active inflammation.

The aim of our study was to verify the potential role of inflammatory markers in the diagnostics of AIH. Serological parameters of liver fibrosis used in our study were presented as additional indices because of the cirrhotic subgroup among investigated patients. Additionally, to the best of our knowledge, the following parameters: NEUT-RI, NEUT-GI, and RE-LYMP were not correlated with MPR, PLR, RPR, RLR, NLR, GPR, AAR, APRI, or FIB-4 in the course of AIH. Especially significant correlations were demonstrated in the subgroup of patients with already developed LC. This gives a new perspective for future investigations and the perception of both inflammation and fibrosis as inseparably connected phenomena among AIH patients developing cirrhosis. However, they are mainly used in various studies, not in everyday clinical practice, and surveys similar to our study might make it possible to finally adapt them for use in everyday hepatology. Nevertheless, we focused on the inflammatory aspect of AIH, not on a fibrotic one. Thus, the correlations found between NEUT-GI and RE-LYMP and the calculated haematological parameters (e.g., NLR, RPR, and RLR) should be highlighted. The determination of the parameters from the EIP group (i.e., NEUT-RI, NEUT-GI, and RE-LYMP) and the systemic inflammatory response (i.e., MPR, RPR, and NLR) in patients with AIH may contribute to the improvement of the diagnostic interpretation of the patient’s examination results. Additionally, the obtained results allow for the conclusion that there is a large potential role of non-invasive haematological parameters in the assessment of liver inflammation.

## 5. Conclusions

In patients with AIH, compared to healthy individuals, higher values of NEUT-RI, NEUT-GI, RE-LYMP, and NLR were observed. These results indicate the potential usefulness of these parameters in the routine diagnosis of AIH. In the conducted study, the following parameters were found to be very useful in terms of diagnostics: EIP (NEUT-RI, NEUT-GI, and RE-LYMP) and systemic inflammatory response markers (MPR, PLR, and RLR) in detecting AIH.

The potential usefulness of the analysed parameters in the diagnosis of AIH is also noteworthy. In patients with LC, statistically significant differences were observed in both the analysed inflammatory response system (RPR, MPR, PLR, WBC, PLT, MPV, NEUT, LYMPH, and MONO) and EIP (NEUT-GI). Particular attention should be paid to the haematological indicators, RPR and MPR, which were characterised by a very high diagnostic usefulness.

According to the achieved data presented here, inflammatory markers seem to be closely related to the serological indices of liver fibrosis, confirming a direct relationship between these phenomena in the course of AIH and AIH-followed liver fibrosis. Observed dependences seem to be a kind of novelty in the field of hepatology. These findings can be perceived as a potential new concept in the diagnosis and monitoring of liver pathologies.

Summing up, the analysed parameters may turn out to be useful in detecting AIH and in looking for features of already developed liver cirrhosis in its course. Nevertheless, at this stage, it is hard to speculate and clarify whether observed deviations in examined inflammatory parameters are related only to AIH or maybe to the general idea of liver disorders. Therefore, the following studies should concern the evaluation of EIP in various liver disorders to compare the obtained results and elucidate their potential status in liver diagnostics.

## Figures and Tables

**Table 1:** Characteristics of the control and study groups.

Demographic Data		
Variable	AIH [*n* = 30]	Control [*n* = 30]
Sex		
Women	27 (90%)	25 (83.33%)
Men	3 (10%)	5 (16.67%)
Age [years] Median (range)	56 (23–80)	43 (21–69)
BMI [kg/m^2^] Median (range)	24.91 (18.67–37.11)	22.60 (17–29.7)
Clinical Data		
Disease duration [years] Median (range)	13 (1–25)	-
Treatment Steroids Immunosuppressive agents Steroids + Immunosuppressive agents	19 (63.33%) 1 (3.33%) 10 (33.34%)	- - -
Family history of AIH Negative Positive	22 (73.33%) 8 (26.67%)	- -
LC Non-LC	10 (33.33%) 20 (66.67%)	- -
Smoking status Smoker Non-smoker	2 (6.67%) 28 (93.33%)	5 (16.67%) 25 (83.33%)
Excessive alcohol consumption Yes No	0 (0%) 30 (100%)	2 (6.67%) 28 (93.33%)
Allergies Yes No	6 (20%) 24 (80%)	- -
Comorbidities Yes * No	15 (50%) 15 (50%)	- -

(AIH—autoimmune hepatitis, BMI—body mass index, LC—liver cirrhosis), * including: diabetes, nephrolithiasis, chronic heart failure, arterial hypertension, osteoporosis, bronchial asthma, and glaucoma.

**Table 2:** Assessment of the usefulness of selected laboratory variables, including Extended Inflammation Parameters as well as calculated indicators—systemic inflammatory response markers and serous indirect markers of liver fibrosis in the diagnosis of AIH.

Variable	Sensitivity (%)	Specificity (%)	Cut-Off	AUC [95%CI]	*p*
RBC [10^6^/µL]	36.67	100	≤4.17	0.64 [0.51–0.76]	0.0495 *
MCV [fl]	76.67	73.33	>88.10	0.81 [0.69–0.90]	<0.0001 *
PLT [10^3^/µL]	40	100	≤163	0.72 [0.59–0.83]	0.0014 *
RDW-SD [fl]	60	100	>45.60	0.84 [0.73–0.92]	<0.0001 *
MPV [fl]	86.67	60	>10.20	0.79 [0.67–0.88]	<0.0001 *
WBC [10^3^/µL]	46.67	93.33	>6.94	0.68 [0.55–0.80]	0.0107 *
NEUT [10^3^/µL]	80	76.67	>3.26	0.78 [0.66–0.88]	<0.0001
LYMPH [10^3^/µL]	63.33	96.67	≤1.49	0.78 [0.66–0.88]	<0.0001 *
MONO [10^3^/µL]	56.67	73.33	>0.54	0.62 [0.48–0.74]	0.1206
IG [10^3^/µL]	66.67	96.67	>0.02	0.79 [0.67–0.89]	<0.0001 *
NEUT-RI [FI]	83.33	73.33	>44.50	0.86 [0.74–0.93]	<0.0001 *
NEUT-GI [SI]	73.33	86.67	>151.10	0.80 [0.68–0.89]	<0.0001 *
AS-LYMP [10^3^/µL]	0	100	>0.00	0.50 [0.37–0.63]	1.0000
RE-LYMP [10^3^/µL]	50	100	>0.07	0.78 [0.66–0.88]	<0.0001 *
CRP [mg/L]	82.76	53.33	>1.50	0.72 [0.58–0.82]	0.0014 *
MPR	66.67	76.67	>0.04	0.77 [0.64–0.87]	<0.0001 *
PLR	100	100	≤36.73	1.00 [0.94–1.00]	<0.0001 *
RPR	56.67	90	>0.06	0.75 [0.63–0.86]	0.0001 *
RLR	100	100	≤2.04	1.00 [0.94–1.00]	<0.0001 *
NLR	70	96.67	>2.37	0.84 [0.72–0.92]	<0.0001 *
GPR	80	96.67	>0.43	0.87 [0.76–0.94]	<0.0001 *
AAR	90	30	≤1.50	0.85 [0.45–0.71]	0.2850
APRI	86.67	96.67	>0.44	0.94 [0.54–0.98]	<0.0001 *
FIB-4	70	86.67	>1.32	0.84 [0.72–0.92]	<0.0001 *

RBC—Red Blood Cells. MCV—Mean Cell Volume. PLT—Platelets. RDW-SD—Red Blood Cell Distribution Width. Standard Deviation. MPV—Mean Platelet Volume. WBC—White Blood Cells. NEUT—Neutrophils. LYMPH—Lymphocytes. MONO—Monocytes. IG—Immature Granulocytes. NEUT-RI—Neutrophil Reactive Intensity. NEUT-GI—Neutrophil Granularity Intensity. AS-LIMPH—Antibody-Secreting Reactive Lymphocytes. RE-LIMPH—Reactive Lymphocytes. CRP—C-Reactive Protein. MPR—Mean Platelet Volume-to-Platelet Ratio. PLR—Platelet-to-Lymphocyte Ratio. RPR—Red Blood Cell Distribution Width-to-Platelet Ratio. RLR—Red Blood Cell Distribution Width-to-Lymphocyte Ratio. NLR—Neutrophil-to-Lymphocyte Ratio. GPR—Gamma-Glutamyl-Transpeptidase-to-Platelet Ratio. AAR—Aspartate Aminotransferase-to-Alanine Aminotransferase Ratio. APRI—Aspartate Aminotransferase-to-Platelet Ratio Index. FIB-4—Fibrosis-4. AUC—Area under curve. * Statistically significant result.

**Table 3:** Assessment of the usefulness of selected laboratory variables, including Extended Inflammation Parameters as well as calculated indicators—systemic inflammatory response markers and serous indirect markers of liver fibrosis in the diagnosis of LC.

Variable	Sensitivity (%)	Specificity (%)	Cut-Off	AUC [95%CI]	*p*
RBC [10^6^/µL]	65	80	>4.32	0.65 [0.46–0.82]	0.1741
MCV [fl]	100	40	>86.70	0.15 [0.37–0.74]	0.6631
PLT [10^3^/µL]	80	100	>201.00	0.94 [0.77–0.99]	<0.0001 *
RDW-SD [fl]	50	80	≤45.60	0.62 [0.43–0.79]	0.2459
MPV [fl]	65	70	≤11.30	0.11 [0.44–0.81]	0.2009
WBC [10^3^/µL]	80	100	>6.20	0.92 [0.76–0.99]	<0.0001 *
NEUT [10^3^/µL]	50	100	<1.84	0.78 [0.59–0.91]	0.0069 *
LYMPH [10^3^/µL]	100	80	>0.91	0.90 [0.73–0.98]	<0.0001 *
MONO [10^3^/µL]	55	100	>0.58	0.79 [0.60–0.92]	0.0006 *
IG [10^3^/µL]	95	50	>0.01	0.77 [0.58–0.90]	0.0072 *
NEUT-RI [FI]	90	50	≤49.50	0.58 [0.39–0.76]	0.5368
NEUT-GI [SI]	65	100	>152.80	0.89 [0.73–0.98]	<0.0001 *
AS-LYMP [10^3^/µL]	0	100	>0.00	0.50 [0.31–0.69]	1.0000
RE-LYMP [10^3^/µL]	85	60	≤0.09	0.68 [0.48–0.84]	0.1550
CRP [mg/L]	60	66.67	≤3.00	0.55 [0.36–0.74]	0.6623
MPR	75	100	≤0.05	0.93 [0.78–0.99]	<0.0001 *
PLR	75	90	>7.28	0.86 [0.68–0.96]	<0.0001 *
RPR	85	90	≤0.08	0.91 [0.75–0.98]	<0.0001 *
RLR	100	30	≤1.37	0.57 [0.38–0.75]	0.5921
NLR	35	50	>3.29	0.50 [0.31–0.69]	1.0000
GPR	25	90	≤0.40	0.51 [0.33–0.70]	0.8993
AAR	50	90	≤0.84	0.73 [0.54–0.88]	0.0172 *
APRI	55	90	≤0.93	0.72 [0.53–0.87]	0.0209 *
FIB-4	60	100	≤1.47	0.83 [0.65–0.94]	<0.0001 *

RBC—Red Blood Cells. MCV—Mean Cell Volume. PLT—Platelets. RDW-SD—Red Blood Cell Distribution Width. Standard Deviation. MPV—Mean Platelet Volume. WBC–White Blood Cells. NEUT—Neutrophils. LYMPH—Lymphocytes. MONO—Monocytes, IG—Immature Granulocytes. NEUT-RI—Neutrophil Reactive Intensity. NEUT-GI—Neutrophil Granularity Intensity. AS-LYMP—Antibody-Secreting Reactive Lymphocytes. RE-LYMP—Reactive Lymphocytes. CRP—C-Reactive Protein. MPR—Mean Platelet Volume-to-Platelet Ratio. PLR—Platelet-to-Lymphocyte Ratio. RPR—Red Blood Cell Distribution Width-to-Platelet Ratio. RLR—Red Blood Cell Distribution Width-to-Lymphocyte Ratio. NLR—Neutrophil-to-Lymphocyte Ratio. GPR—Gamma-Glutamyl-Transpeptidase-to-Platelet Ratio. AAR—Aspartate Aminotransferase–to-Alanine Aminotransferase Ratio. APRI—Aspartate Aminotransferase-to-Platelet Ratio Index. FIB-4—Fibrosis-4. AUC—Area under curve. * Statistically significant result.

## Data Availability

The data used in this study are sensitive patient data, partly belonging to Independent Public Clinical Hospital No. 4 in Lublin. For this reason, they can only be shared with the consent of the hospital. In this case, please contact the hospital’s data protection officer or the director of the hospital for medical affairs (ul. Jaczewskiego 8, 20-954 Lublin, szpital@spsk4.lublin.pl).

## Supplementary Materials

The following supporting information can be downloaded at: https://www.mdpi.com/article/10.3390/cells11162554/s1, Table S1: Comparison of selected laboratory variables, including Extended Inflammation Parameters as well as calculated indicators—systemic inflammatory response markers and serous indirect markers of liver fibrosis in both the control and study groups; Table S2: The correlation between EIP and systemic inflammatory response markers and serous indirect markers of liver fibrosis in the study group (AIH); Table S3: The correlation between EIP and systemic inflammatory response markers and serous indirect markers of liver fibrosis in the AIH-non-LC group; Table S4: The correlation between EIP and systemic inflammatory response markers and serous indirect markers of liver fibrosis in the AIH-LC group; Table S5: Comparison of selected laboratory variables, including Extended Inflammation Parameters as well as calculated indicators—systemic inflammatory response markers and serous indirect markers of liver fibrosis in the LC and non-LC groups; Table S6: Comparison of selected laboratory variables, including Extended Inflammation Parameters as well as calculated indicators—systemic inflammatory response markers and serous indirect markers of liver fibrosis depending on the applied treatment.
