# Supplementary material for: The Usefulness of Extended Inflammation Parameters and Systemic Inflammatory Response Markers in the Diagnostics of Autoimmune Hepatitis
Source: Cells. 2022 Aug 17;11(16):2554. doi: 10.3390/cells11162554 (PMC9406892; doi:10.3390/cells11162554)
Supplement: Supplementary file 1 [file cells-11-02554-s001.zip › cells-1823921-supplementary.pdf]

Supplementary Table S1. Comparison of selected laboratory variables, including Extended Inflammation Parameters as well as calculated indicators – systemic inflammatory response markers and serous indirect markers of liver fibrosis in both the control and study groups.

**Variable**	**Control [*n* = 30] Median (Interquartile Range)**	**AIH [*n* = 30] Median (Interquartile Range)**	***p***
RBC [$10^6/\mu L$]	4.52 (4.27–4.77)	4.32 (4.02–4.55)	0.0555
MCV [fl]	87.20 (84.80–88.60)	92.55 (88.20–94.5)	<0.0001 *
PLT [$10^3/\mu L$]	265.5 (217–316)	218 (89–255)	0.0038 *
RDW-SD [fl]	41.27 (40–43)	46.40 (42.50–51.80)	0.0013 *
MPV [fl]	10.10 (9.70–10.80)	11.30 (10.70–12.70)	0.0001 *
WBC [$10^3/\mu L$]	5.58 (4.83–6.50)	6.47 (5.42–8.75)	0.0147 *
NEUT [$10^3/\mu L$]	2.74 (2.32–3.26)	4.53 (3.40–6.79)	0.0002 *
LYMPH [$10^3/\mu L$]	1.83 (1.70–2.20)	1.41 (0.91–1.86)	0.0001 *
MONO [$10^3/\mu L$]	0.43 (0.35–0.58)	0.57 (0.43–0.68)	0.1135
IG [$10^3/\mu L$]	0.01 (0.01–0.02)	0.03 (0.02–0.06)	0.0001 *
NEUT-RI [FI]	43.30 (42.60–45.20)	48.05 (45–49.5)	<0.0001 *
NEUT-GI [SI]	147.40 (144.00–150.10)	152.65 (150.5–156.20)	0.0001 *
AS-LYMP [$10^3/\mu L$]	0 (0–0)	0 (0–0)	1.0000
RE-LYMP [$10^3/\mu L$]	0.03 (0.03–0.06)	0.07 (0.04–0.10)	0.0001 *
CRP [mg/l]	1.5 (1–3)	3 (2.00–5.15)	0.0043 *
MPR	0.04 (0.03–0.04)	0.05 (0.04–0.14)	0.0004 *
PLR	137.37 (104.45–168.18)	8.5 (6.59–11.36)	<0.0001 *
RPR	0.05 (0.04–0.06)	0.07 (0.05–0.15)	0.0007 *
RLR	7.17 (5.95–7.47)	0.61 (0.46–1.18)	<0.0001 *
NLR	1.42 (1.16–1.85)	2.81 (1.95–5.85)	<0.0001 *
AST [IU/L]	21.50 (18.00–26)	54 (39–105)	<0.0001 *
ALT [IU/L]	18.00 (12–28)	62.5 (34–148)	<0.0001 *
Bilirubin [mg/dl]	0.60 (0.40–0.80)	1.50 (0.60–1.80)	0.0002 *
ALP [IU/L]	68 (62–98)	128 (0.79–190)	0.0002 *
GGTP [IU/L]	18 (13–27)	100.50 (44–208)	<0.0001 *
GPR	0.18 (0.12–0.23)	1.78 (0.74–3.90)	<0.0001 *
AAR	1.13 (0.86–1.54)	1.04 (0.74–1.36)	0.2836
APRI	0.27 (0.18–0.31)	1.37 (0.49–2.52)	<0.0001 *
FIB-4	0.83 (0.51–1.14)	1.82 (1.11–4.24)	<0.0001 *

RBC—Red Blood Cells. MCV—Mean Cell Volume. PLT—Platelets. RDW-SD—Red Blood Cell Distribution Width. Standard Deviation. MPV—Mean Platelet Volume. WBC—White Blood Cells. NEUT—Neutrophils. LYMPH—Lymphocytes. MONO—Monocytes. IG—Immature Granulocytes. NEUT-RI—Neutrophil Reactive Intensity. NEUT-GI—Neutrophil Granularity Intensity. AS-LIMPH—Antibody-Secreting Reactive Lymphocytes. RE-LIMPH—Reactive Lymphocytes. CRP—C-Reactive Protein. MPR—Mean Platelet Volume-to-Platelet Ratio. PLR—Platelet-to-Lymphocyte Ratio. RPR—Red Blood Cell Distribution Width-to-Platelet Ratio. RLR—Red Blood Cell Distribution Width-to-Lymphocyte Ratio. NLR—Neutrophil-to-Lymphocyte Ratio. AST—Aspartate Aminotransferase. ALT—Alanine Aminotransferase. ALP–Alkaline Phosphatase. GGTP—Gamma-Glutamyl Transpeptidase. GPR—Gamma-Glutamyl-Transpeptidase-to-Platelet Ratio. AAR—Aspartate Aminotransferase–to-Alanine Aminotransferase Ratio. APRI—Aspartate Aminotransferase-to-Platelet Ratio Index. FIB-4—Fibrosis-4. * Statistically significant result.

Supplementary Table S2. The correlation between EIP and systemic inflammatory response markers and serous indirect markers of liver fibrosis in the study group (AIH).

Variable	NEUT-RI [FI]		NEUT-GI [SI]		RE-LYMP [$10^3/\mu L$]	
	**Rho**	***p***	**Rho**	***p***	**rho**	***p***
MPR	0.066	0.7285	–0.493	0.0056 *	–0.017	0.9284
PLR	0.027	0.8886	0.143	0.4507	0.215	0.2549
RPR	0.020	0.9168	–0.477	0.0077 *	–0.022	0.9070
RLR	0.130	0.4929	–0.144	0.4486	0.255	0.1746
NLR	0.132	0.4876	0.007	0.9693	0.021	0.9122
GPR	0.322	0.0829	0.002	0.9898	0.211	0.2621
AAR	0.138	0.4672	–0.319	0.0862	0.037	0.8474
APRI	0.313	0.0923	–0.310	0.0951	0.193	0.3073
FIB-4	0.101	0.5936	–0.659	0.0001 *	0.064	0.7361

NEUT-RI—Neutrophil Reactive Intensity. NEUT-GI—Neutrophil Granularity Intensity. RE-LIMPH—Reactive Lymphocytes. MPR—Mean Platelet Volume-to-Platelet Ratio. PLR—Platelet-to-Lymphocyte Ratio. RPR—Red Blood Cell Distribution Width-to-Platelet Ratio. RLR—Red Blood Cell Distribution Width-to-Lymphocyte Ratio. NLR—Neutrophil-to-Lymphocyte Ratio. GPR—Gamma-Glutamyl-Transpeptidase-to-Platelet Ratio. AAR—Aspartate Aminotransferase-to-Alanine Aminotransferase Ratio. APRI—Aspartate Aminotransferase-to-Platelet Ratio Index. FIB-4—Fibrosis-4. *Statistically significant result.

Supplementary Table S3. The correlation between EIP and systemic inflammatory response markers and serous indirect markers of liver fibrosis in the AIH-non-LC group.

Variable	NEUT-RI [FI]		NEUT-GI [SI]		RE-LYMP [$10^3/\mu L$]	
	**rho**	***P***	**rho**	***p***	**rho**	***p***
**MPR**	–0.058	0.7968	–0.274	0.2169	–0.151	0.5019
**PLR**	0.319	0.1482	–0.239	0.2831	0.641	0.0013 *
**RPR**	–0.152	0.4981	–0.298	0.1776	–0.172	0.4429
**RLR**	0.243	0.2758	–0.264	0.2345	0.392	0.0708
**NLR**	0.311	0.1584	0.008	0.9702	0.150	0.5055
**GPR**	0.135	0.5507	0.052	0.8184	0.092	0.6854
**AAR**	0.196	0.3832	–0.202	0.3682	0.038	0.8675
**APRI**	0.347	0.1136	–0.226	0.3108	0.247	0.2675
**FIB-4**	–0.027	0.9046	–0.519	0.0133 *	0.039	0.8615

NEUT-RI—Neutrophil Reactive Intensity. NEUT-GI—Neutrophil Granularity Intensity. RE-LYMP—Reactive Lymphocytes. MPR—Mean Platelet Volume-to-Platelet Ratio. PLR—Platelet-to-Lymphocyte Ratio. RPR—Red Blood Cell Distribution Width-to-Platelet Ratio. RLR—Red Blood Cell Distribution Width-to-Lymphocyte Ratio. NLR—Neutrophil-to-Lymphocyte Ratio. GPR—Gamma-Glutamyl-Transpeptidase-to-Platelet Ratio. AAR—Aspartate Aminotransferase-to-Alanine Aminotransferase Ratio. APRI—Aspartate Aminotransferase-to-Platelet Ratio Index. FIB-4—Fibrosis-4. * Statistically significant result.

Supplementary Table S4. The correlation between EIP and systemic inflammatory response markers and serous indirect markers of liver fibrosis in the AIH-LC group.

Variable	NEUT-RI [FI]		NEUT-GI [SI]		RE-LYMP [10^3/μL]	
	rho	*p*	rho	*P*	rho	*p*
**MPR**	0.357	0.3851	0.238	0.5702	0.214	0.6103
**PLR**	–0.119	0.7789	–0.119	0.7789	–0.262	0.5309
**RPR**	0.357	0.3851	0.238	0.5702	0.214	0.6103
**RLR**	0.238	0.5702	0.119	0.7789	–0.048	0.9108
**NLR**	0.190	0.6514	–0.048	0.9108	–0.095	0.8225
**GPR**	0.690	0.0580	0.048	0.9108	0.548	0.1600
**AAR**	–0.048	0.9108	–0.238	0.5702	–0.143	0.7358
**APRI**	0.095	0.8225	0.286	0.4927	–0.095	0.8225
**FIB-4**	0.286	0.4927	–0.357	0.3851	–0.095	0.8225

NEUT-RI—Neutrophil Reactive Intensity. NEUT-GI—Neutrophil Granularity Intensity. RE-LYMP—Reactive Lymphocytes. MPR—Mean Platelet Volume-to-Platelet Ratio. PLR—Platelet-to-Lymphocyte Ratio. RPR– Red Blood Cell Distribution Width-to-Platelet Ratio. RLR—Red Blood Cell Distribution Width-to-Lymphocyte Ratio. NLR—Neutrophil-to-Lymphocyte Ratio. GPR—Gamma-Glutamyl-Transpeptidase-to-Platelet Ratio. AAR—Aspartate Aminotransferase-to-Alanine Aminotransferase Ratio. APRI—Aspartate Aminotransferase-to-Platelet Ratio Index. FIB-4—Fibrosis-4.

Supplementary Table S5. Comparison of selected laboratory variables, including Extended Inflammation Parameters as well as calculated indicators – systemic inflammatory response markers and serous indirect markers of liver fibrosis in the LC and non-LC groups.

Variable	LC [*n* = 10] Median (Interquartile Range)	non-LC [*n* = 20] Median (Interquartile Range)	*p*
RBC [10^6/μL]	4.20 (3.82–4.32)	4.45 (4.06–4.56)	0.1726
MCV [fl]	89.30 (85.50–101.60)	92.80 (89.50–94.40)	0.5673
PLT [10^3/μL]	85(59–134)	241.50 (218.00–299.50)	0.0001 *
RDW-SD [fl]	48.80 (46.30–51.80)	45.95 (61.10–41.65)	0.2811
MPV [fl]	11.65 (10.70–12.90)	11.10 (10.60–12.15)	0.2263
WBC [10^3/μL]	4.94 (2.48–6.13)	7.75 (6.47–9.97)	0.0002 *
NEUT [10^3/μL]	3.52 (1.32–4.76)	4.91 (3.97–7.80)	0.0114 *
LYMPH [10^3/μL]	0.86 (0.80–0.91)	1.54 (1.35–1.95)	0.0005 *
MONO [10^3/μL]	0.43 (0.25–0.58)	0.66 (0.47–0.90)	0.0114 *
IG [10^3/μL]	0.02 (0.00–0.05)	0.04 (0.03–0.09)	0.0197 *
NEUT-RI [FI]	47.95 (45.00–56.20)	48.05 (46.45–48.70)	0.4679
NEUT-GI [SI]	148.15 (144.30–152.10)	155 (152.50–158.45)	0.0006 *
AS-LYMP [10^3/μL]	0 (0–0)	0 (0–0)	0.9825
RE-LYMP [10^3/μL]	0.10 (0.05–0.12)	0.06 (0.04–0.09)	0.1108
CRP [mg/L]	3.20 (2.27–5.60)	3 (2.00–4.60)	0.6714
MPR	0.15 (0.08–0.20)	0.04 (0.04–0.05)	0.0002 *
PLR	4.37 (2.42–7.28)	10.06 (7.52–14.96)	0.0017 *
RPR	0.16 (0.11–0.26)	0.06 (0.04–0.07)	0.0003 *
RLR	0.73 (0.46–1.85)	0.57 (0.48–1.18)	0.5526
NLR	3.28 (1.59–5.94)	2.77 (1.99–5.85)	0.9825
GPR	1.85 (0.86–3.58)	1.78 (0.57–3.94)	0.9124
AAR	1.22 (0.95–1.96)	0.88 (0.66–1.26)	0.0408 *
APRI	2.01 (1.37–3.64)	0.79 (0.46–1.99)	0.0557
FIB-4	3.59 (1.97–10.38)	1.46 (0.93–1.99)	0.0034 *

RBC—Red Blood Cells. MCV—Mean Cell Volume. PLT—Platelets. RDW-SD—Red Blood Cell Distribution Width. Standard Deviation. MPV—Mean Platelet

Volume. WBC—White Blood Cells. NEUT—Neutrophils. LYMPH – Lymphocytes. MONO—Monocytes, IG—Immature Granulocytes. NEUT-RI–Neutrophil Reactive Intensity. NEUT-GI—Neutrophil Granularity Intensity. AS-LYMP—Antibody-Secreting Reactive Lymphocytes. RE-LYMP—Reactive Lymphocytes. CRP—C-Reactive Protein. MPR—Mean Platelet Volume-to-Platelet Ratio. PLR—Platelet-to-Lymphocyte Ratio. RPR—Red Blood Cell Distribution Width-to-Platelet Ratio. RLR—Red Blood Cell Distribution Width-to-Lymphocyte Ratio. NLR—Neutrophil-to-Lymphocyte Ratio. GPR—Gamma-Glutamyl-Transpeptidase-to-Platelet Ratio. AAR—Aspartate Aminotransferase–to-Alanine Aminotransferase Ratio. APRI—Aspartate Aminotransferase-to-Platelet Ratio Index. FIB-4—Fibrosis-4. *Statistically significant result.

Supplementary Table S6. Comparison of selected laboratory variables, including Extended Inflammation Parameters as well as calculated indicators – systemic inflammatory response markers and serous indirect markers of liver fibrosis depending on the applied treatment.

Variable	No Drug [A]	S Alone [B]	S + IS Agents [C]	S Alone or S + IS Agents [D]	A vs. B *p*	A vs. C *p*	A vs. D *p*
	Median (Interquartile Range)						
RBC [10^6/μL]	4.22 (3.60–4.52)	4.33 (4.02–4.58)	4.33 (4.02–4.41)	4.34 (4.11–4.58)	0.7333	0.3571	0.3911
MCV [fl]	93.55 (89.30–98.90)	90.80 (85.50–94.50)	91.80 (85.50–94.50)	91.75 (88.20–94.90)	0.4535	0.8096	0.5362
PLT [10^3/μL]	205.50 (103.00–253.50)	222.00 (89.00–283.00)	231.50 (89.00–283.00)	221.00 (143.00–83.00)	0.7333	0.8096	0.7464
RDW-SD [fl]	48.45 (42.55–56.35)	46.50 (42.50–55.80)	48.20 (43.20–55.80)	46.40 (41.90–51.80)	0.7333	0.7363	0.9298
MPV [fl]	11.55 (11.45–12.50)	11.30 (10.60–11.90)	11.35 (10.70–11.90)	11.30 (10.60–12.60)	0.1878	0.4690	0.2448
WBC [10^3/μL]	5.62 (4.86–6.78)	6.96 (4.28–8.75)	7.01 (6.13–8.75)	6.84 (5.42–9.05)	0.3736	0.4690	0.4607
NEUT [10^3/μL]	4.02 (2.93–4.79)	4.39 (1.84–6.79)	4.41 (3.69–6.79)	4.41 (3.33–6.17)	0.6354	0.4690	0.5760
LYMPH [10^3/μL]	1.17 (0.86–1.64)	1.49 (1.03–2.09)	1.48 (1.03–2.09)	1.54 (1.31–1.95)	0.4535	0.2621	0.2708
MONO [10^3/μL]	0.52 (0.49–0.56)	0.57 (0.31–0.68)	0.58 (0.31–0.68)	0.58 (0.33–0.81)	0.7333	0.8096	0.7022
IG [10^3/μL]	0.04 (0.03–0.05)	0.03 (0.02–0.05)	0.03 (0.02–0.04)	0.03 (0.02–0.06)	0.5035	0.8854	0.9756
NEUT-RI [FI]	52.95 (50.05–56.50)	47.90 (46.20–50.10)	47.45 (46.20–49.50)	47.00 (44.80–48.80)	0.0240 *	0.0062 *	0.0088 *
NEUT-GI [SI]	150.40 (147.05–155.60)	152.50 (149.30–157.60)	152.75 (151.10–157.60)	152.50 (150.50–156.00)	0.3736	0.5304	0.4979
AS-LYMP [10^3/μL]	0.00 (0.00–0.00)	0.00 (0.00–0.00)	0.00 (0.00–0.00)	0.00 (0.00–0.00)	0.9436	0.9601	0.9757
RE-LYMP [10^3/μL]	0.11 (0.09–0.13)	0.10 (0.04–0.15)	0.10 (0.04–0.11)	0.09 (0.04–0.11)	0.7333	0.1522	0.3911
CRP [mg/l]	4.10 (3.10–7.23)	2.67 (2.00–4.00)	2.84 (2.00–4.00)	3.00 (2.00–4.20)	0.1419	0.6646	0.2985
MPR	0.06 (0.05–0.18)	0.05 (0.04–0.14)	0.05 (0.04–0.14)	0.05 (0.04–0.08)	0.5395	0.5304	0.4979
PLR	11.92 (6.86–62.87)	6.95 (5.09–11.37)	8.50 (6.59–11.37)	9.22 (6.75–12.44)	0.5395	0.6646	0.5362
RPR	0.07 (0.06–0.32)	0.07 (0.05–0.18)	0.07 (0.05–0.18)	0.07 (0.05–0.11)	0.8392	0.5304	0.6169
RLR	1.42 (0.68–5.28)	0.58 (0.42–1.18)	0.60 (0.46–1.18)	0.58 (0.42–1.18)	0.2398	0.1847	0.1392
NLR	3.07 (2.21–4.75)	2.49 (1.59–4.63)	2.59 (1.95–4.63)	2.58 (1.70–3.88)	0.7333	0.6646	0.5760
AST [IU/L]	52.00 (48.00–55.50)	55.00 (28.00–204.00)	49.00 (28.00–204.00)	56.00 (38.00–152.00)	0.9451	0.8096	0.8370
ALT [IU/L]	34.00 (30.50–45.50)	65.00 (28.00–148.00)	72.00 (28.00–148.00)	78.00 (47.00–148.00)	0.3037	0.0624	0.0820
Bilirubin [mg/dl]	0.90 (0.65–2.25)	1.00 (0.50–4.70)	1.10 (0.70–4.70)	1.25 (0.70–1.80)	0.7333	0.5965	0.7022
ALP [IU/L]	119.00 (72.00–144.50)	143.00 (74.00–217.00)	146.50 (74.00–217.00)	135.50 (79.00–217.00)	0.4535	0.5304	0.4979
GGTP [IU/L]	96.00 (52.50–330.00)	122.00 (14.00–228.00)	127.00 (14.00–228.00)	117.00 (44.00–213.00)	1.0000	1.0000	1.0000
GPR	2.45 (0.80–6.19)	2.27 (0.15–3.97)	2.40 (0.15–3.97)	1.78 (0.44–3.90)	0.8392	0.5965	0.6169
AAR	1.41 (1.17–1.73)	0.95 (0.72–1.39)	0.96 (0.72–1.39)	0.91 (0.72–1.26)	0.1419	0.0196 *	0.0262 *
APRI	0.91(0.61–2.41)	1.04 (0.45–6.23)	1.52 (0.45–6.23)	1.21 (0.49–2.35)	1.0000	0.8854	0.9298
FIB-4	1.99 (1.17–10.01)	1.97 (1.45–3.13)	1.96 (1.45–3.13)	1.96 (1.21–2.70)	0.9451	0.7363	0.7912

S—steroids. IS—immunosuppressive agents. RBC—Red Blood Cells. MCV—Mean Cell Volume. PLT—Platelets. RDW-SD—Red Blood Cell Distribution Width. Standard Deviation. MPV—Mean Platelet Volume. WBC—White Blood Cells. NEUT—Neutrophils. LYMPH—Lymphocytes. MONO—Monocytes. IG—Immature Granulocytes. NEUT-RI—Neutrophil Reactive Intensity. NEUT-GI—Neutrophil Granularity Intensity. AS-LIMPH—Antibody-Secreting Reactive Lymphocytes. RE-LIMPH—Reactive Lymphocytes. CRP—C-Reactive Protein. MPR—Mean Platelet Volume-to-Platelet Ratio. PLR—Platelet-to-Lymphocyte Ratio. RPR—Red Blood Cell Distribution Width-to-Platelet Ratio. RLR—Red Blood Cell Distribution Width-to-Lymphocyte Ratio. NLR—Neutrophil-to-Lymphocyte Ratio. AST—Aspartate Aminotransferase. ALT—Alanine Aminotransferase. ALP–Alkaline Phosphatase. GGTP—Gamma-Glutamyl Transpeptidase. GPR—Gamma-Glutamyl-Transpeptidase-to-Platelet Ratio. AAR—Aspartate Aminotransferase–to-Alanine Aminotransferase Ratio. APRI—Aspartate Aminotransferase-to-Platelet Ratio Index. FIB-4—Fibrosis-4. * Statistically significant result.
